# Characterization of increasing stages of invasiveness identifies stromal/cancer cell crosstalk in rat models of mesothelioma

**DOI:** 10.18632/oncotarget.24632

**Published:** 2018-03-27

**Authors:** Joëlle S. Nader, Jérôme Abadie, Sophie Deshayes, Alice Boissard, Stéphanie Blandin, Christophe Blanquart, Nicolas Boisgerault, Olivier Coqueret, Catherine Guette, Marc Grégoire, Daniel L. Pouliquen

**Affiliations:** ^1^ CRCINA, INSERM, Université d’Angers, Université de Nantes, Nantes, France; ^2^ ONIRIS, Nantes, France; ^3^ CRCINA, INSERM, Université de Nantes, Université d’Angers, Angers, France; ^4^ ICO, Angers, France; ^5^ Plate-Forme MicroPICell, SFR François Bonamy, Université de Nantes, France

**Keywords:** stroma, invasiveness, sarcomatoid mesothelioma, immune cells, rat model

## Abstract

Sarcomatoid mesothelioma (SM) is a devastating cancer associated with one of the poorest outcome. Therefore, representative preclinical models reproducing different tumor microenvironments (TME) observed in patients would open up new prospects for the identification of markers and evaluation of innovative therapies. Histological analyses of four original models of rat SM revealed their increasing infiltrative and metastatic potential were associated with differences in Ki67 index, blood-vessel density, and T-lymphocyte and macrophage infiltration. In comparison with the noninvasive tumor M5-T2, proteomic analysis demonstrated the three invasive tumors F4-T2, F5-T1 and M5-T1 shared in common a very significant increase in the abundance of the multifunctional proteins galectin-3, prohibitin and annexin A5, and a decrease in proteins involved in cell adhesion, tumor suppression, or epithelial differentiation. The increased metastatic potential of the F5-T1 tumor, relative to F4-T2, was associated with an increased macrophage vs T-cell infiltrate, changes in the levels of expression of a panel of cytokine genes, an increased content of proteins involved in chromatin organization, ribosome structure, splicing, or presenting anti-adhesive properties, and a decreased content of proteins involved in protection against oxidative stress, normoxia and intracellular trafficking. The most invasive tumor, M5-T1, was characterized by a pattern of specific phenotypic and molecular features affecting the presentation of MHC class I-mediated antigens and immune cell infiltration, or involved in the reorganization of the cytoskeleton and composition of the extracellular matrix. These four preclinical models and data represent a new resource available to the cancer research community to catalyze further investigations on invasiveness.

## INTRODUCTION

For cancers located in deep cavities, early symptoms are often absent, and thus the majority of patients are diagnosed late in the disease, leading to very low five-year survival rates. For such patients, a better understanding of the crosstalk between tumor cells and components of the tumor microenvironment (TME) (stromal cells including immune cells, blood vessels, extracellular matrix (ECM), and associated signaling molecules) [1] is critical.

Stromal cells are involved in growth promotion, local invasion and metastatic spread. Recent data have provided evidence that during cancer progression, an interactive relationship occurs between metastatic cancer cells and the host stroma, these dynamic interactions influencing the metastatic process [2]. Stromal cells are also involved in immune escape. The last two decades of immuno-oncology research have demonstrated that tumor development can be stopped or controlled through a process known as immunosurveillance [3]. The current clinical success of anticancer immunotherapy is encouraging but considerable uncertainties remain, as less than half of all patients show objective antitumor responses. This raises the question of what else may be required to improve success rates [4]. In fact, an increased number of tumor-specific cytotoxic T lymphocytes does not correlate with clinical efficacy [5]. Thus, antitumor effector T-cell quantity or quality may not be the key, as the TME can confer profound resistance to lymphocyte-induced cell death [4]. Recent findings in this field have demonstrated that CD8+ T cells recognizing tumor-specific antigens detected in patients are dysfunctional early in the tumorigenic process [6], while the TME contributes to the exclusion of T cells from the vicinity of cancer cells, leading to their concentration outside the tumor field [7].

Regarding the role of myeloid cells in tumors, evidence indicates that the TME alters myeloid cells by converting them into potent immunosuppressive cells [8, 9]. Due the emergence of new tools, in this context the individual contributions of the different subsets of monocytes, macrophages, and DCs has started to be clarified [10]. In particular, recent findings have demonstrated that the differentiation of tumor-associated macrophages (TAMs) from monocytic precursors at tumor sites is controlled by downregulation of the activity of the STAT3 transcription factor induced by cancer cells [11]. In parallel, as increased tumor growth was previously observed in rats bearing peritoneal metastatic tumors with decreased macrophage recruitment [12], some important questions remain regarding the versatility and plasticity of cells of the monocyte–macrophage lineage [13], emphasizing the need to improve our understanding of their mechanics of activation in different microenvironments [14].

In this context, interactions between TME components and tumor cells were investigated in four models of experimental malignant mesotheliomas exhibiting marked differences in their proliferation indexes and infiltrative and metastatic potentials [15]. Phenotypic investigations of tumor stroma, combined with SWATH-MS, a new advanced and robust technique allowing the study of proteomic diversity in a quantitative manner in tissues [16], and analysis of the expression levels of cytokines and growth factors in frozen tumor samples, allowed the identification of three stages of increasing invasiveness, characterized by specific cellular and molecular features.

## RESULTS

Four models of rat experimental malignant mesotheliomas were investigated, named M5-T2, F4-T2, F5-T1, and M5-T1 according to the original primary cell lines from which they originated [15]. Overall, the main differences observed between the four models, which correspond to increasing stages of invasiveness of sarcomatoid mesothelioma tumors, are summarized in Table 1.

**Table 1:** Summary of the main differences found between the different models, representing increasing stages of invasiveness in sarcomatoid mesothelioma tumors

	Proliferation	Infiltration of abdominal organs	Leukocytic infiltrate	Proteomic profile	Cytokine expression
**M5-T2**	Moderate	No	+	Multifunctional proteins (low) Adhesion, tumor suppressor (high)	-
**F4-T2**	High	Mild (periphery)	+++	IFM3 transmembrane protein (low) Cytosolic aminopeptidase (high) Immunoreactivity	*Ifng, Ccl5, Cxcl10 (high)*
**F5-T1**	High	Moderate (some intraparenchymal metastases)	++	C-type mannose receptor 2 (high) Inflammatory reaction	*Ccl2, Ccl7, Cxcl1 (high)*
**M5-T1**	High	Deep (multiple metastases)	+/-	Transmembrane proteins (high) ECM, cytoskeletal proteins (high) Immunosuppression	*Ifng, Ccl2, Ccl7, Ccl5, Cxcl10, Ccl11* (low) *Fgf2* (high)

### Histological and immunohistochemical investigations of cancer and stromal cell interactions

**Figure 1:**
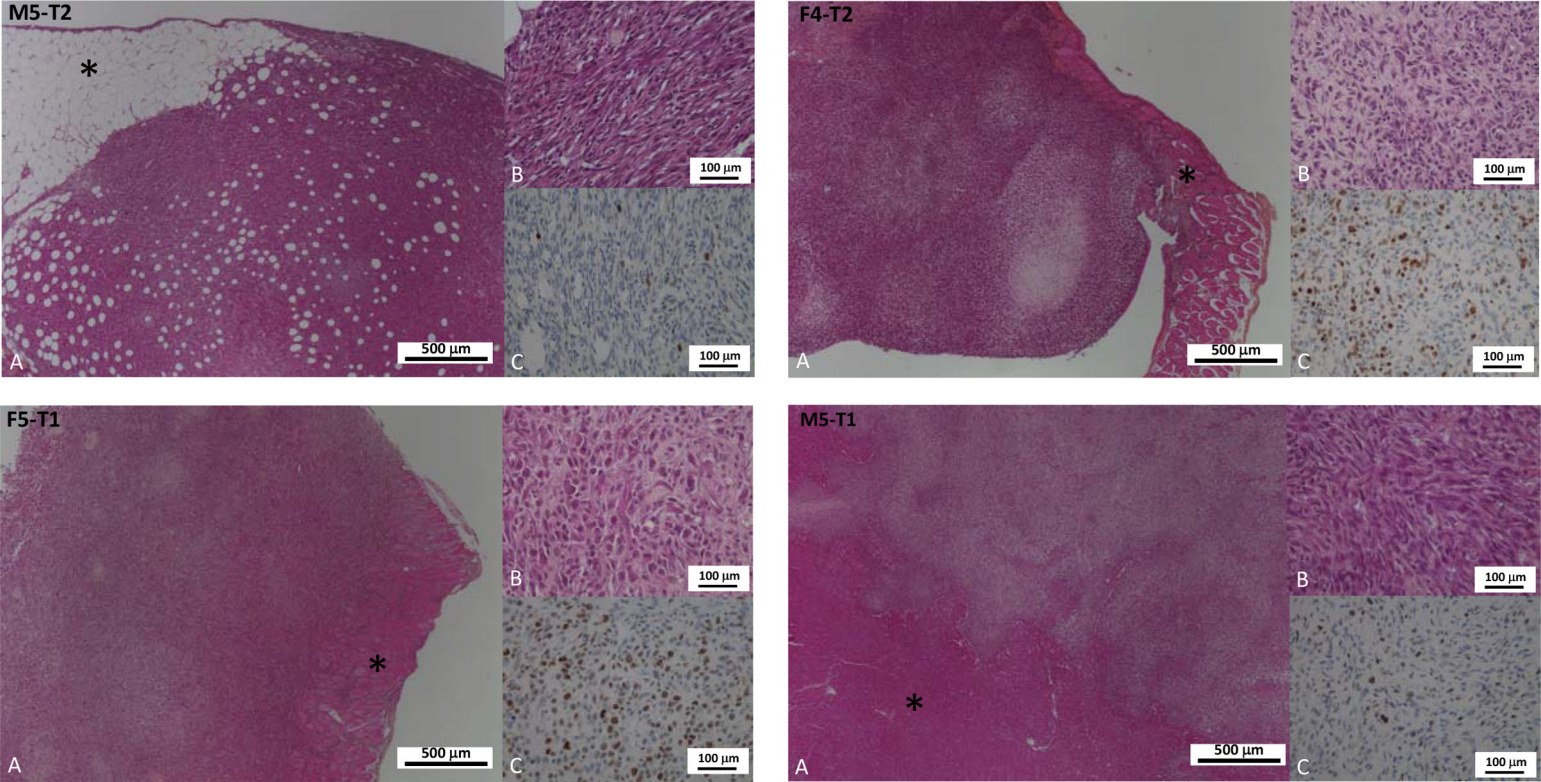
Distinctive features of cancer cell phenotypes of the four models of experimental malignant mesotheliomas **(A** and **B)**: Hematoxylin-phloxine-saffron (HPS) staining (respectively low and high magnification); **(C)**: Immunohistochemical detection of the Ki67 antigen (proliferation index). The four models displayed distinctive characteristics: the M5-T2 tumors are located strictly on the omental serosal surface (asterisk: connective adipose tissue and composed of monomorphic neoplastic cells with moderate atypias and low proliferative activity (C: low number of immunopositive nuclei); the F4-T2 and F5-T1 neoplasms are characterized by increased infiltrative potential with invasion of the deep muscular layers (diaphragm and abdominal wall: asterisk) and are composed of cells with marked atypias and very high ki67 index of proliferation (C: high number of immunopositive nuclei); the M5-T1 tumors are associated with deep infiltration of abdominal organs (liver parenchyma on the picture: asterisk), marked atypias and moderate to high proliferation (C: intermediate number of immunopositive nuclei).

**Figure 2:**
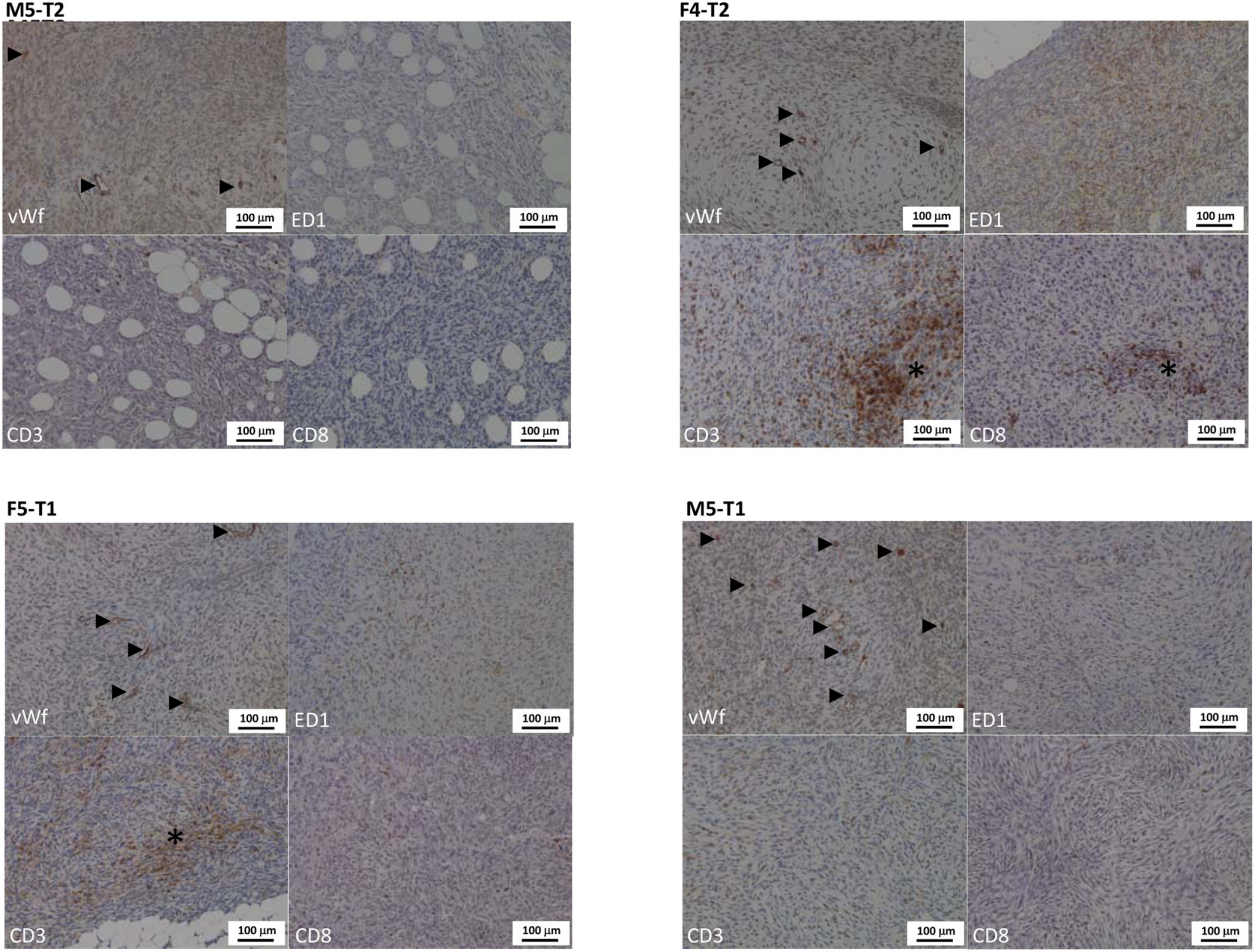
Distinctive features of the vascular and immune stroma of the four models of experimental malignant mesotheliomas Immunohistochemical evaluation of blood vessels density and size (vWf: von Willebrand factor), and of the macrophagic (ED1) and T-lymphocytic (CD3 and CD8) infiltration of the tumor stroma. The four models displayed distinctive characteristics: the M5-T2 tumors display a moderate blood vessel density (arrowheads) and a low T lymphocytic and macrophagic infiltration of the stroma (very low number of immunopositive cells on the ED1, CD3 and CD8 pictures); the F4-T2 and F5-T1 neoplasms are characterized by a moderate angiogenic activity (arrowheads) and a marked to severe infiltration of the tumor stroma by macrophages and particularly CD3+ T-lymphocytes, including CD8+ T-cells (asterisks: high number of immunopositive cells on the ED1, CD3 and CD8 pictures); the M5-T1 tumors are characterized by a high vascular density (arrowheads) and a low T lymphocytes and macrophages infiltration, particularly of the center of the nodules, with immune cells mostly located at the periphery of the neoplastic tissue (low number of immunopositive cells on the ED1, CD3 and CD8 pictures).

Histological and phenotypical features of the four models are depicted and described, respectively, in Table 2 and Figure 1. After intraperitoneal injection of these different cell lines, all four models were characterized by multiple nodules disseminated on the omentum and serosal surfaces of the abdominal viscera. The nodules were mostly characterized by a sarcomatoid morphology, with solid sheets and bundles of pleomorphic spindle cells. The M5-T2 tumor differed from the three others by the absence of infiltrative potential, with tumor cell development restricted to the serosal surface, without capsular breakthrough. The cells displayed moderate cytonuclear atypias and moderate proliferative activity, with a mean Ki67 index of 6 per high-power field (HPF). The tumor stroma displayed a low blood-vessel density and a low-to-moderate level of T-lymphocyte and macrophage infiltration, either at the periphery or in the vicinity of tumor cells (Figure 2).

**Table 2:** Histological and immunohistochemical features of cancer and stromal cell interactions

	Histological type^*^	Infiltration	Atypia	Mean Ki67 index per 10 HPF	Blood vessel density	CD3+ T-cell count (tumor periphery) per HPF	CD3+ T-cell count (tumor center) per HPF	CD8+ T-cell count (tumor periphery) per HPF	CD8+ T-cell count (tumor center) per HPF	ED1+ macrophages (tumor periphery) per HPF	ED1+ macrophages (tumor center) per HPF
**M5-T2**	Sarcomatoid	-	+/-	56	+/-	36	96	48	82	20	36
	Sarcomatoid	-	+/-	61	+/-	28	82	18	48	10	32
	**Mean**			**58.5**		**32.0**	**89.0**	**33.0**	**65.0**	**15.0**	**34.0**
**F4-T2**	Mixed, mainly sarcomatoid	+/-	+	248	+	88	62	64	56	64	22
	Sarcomatoid	+/-	+/-	164	+	92	132	88	62	32	110
	Sarcomatoid	+/-	+	148	+	98	56	110	64	48	88
	**Mean**			**186.7**		**92,7**	**83.3**	**87.3**	**60.7**	**48.0**	**73.3**
**F5-T1**	Sarcomatoid	+	+	180	+/-	46	88	46	52	52	96
	Mixed, mainly sarcomatoid	+	++	220	+	32	44	12	56	28	82
	Sarcomatoid	+	+	186	+	28	56	14	36	62	52
	Mixed, mainly sarcomatoid	++	+	228	+	36	46	28	30	30	18
	**Mean**			**203.5**		**35.5**	**58.5**	**25.0**	**43.5**	**43.0**	**62.0**
**M5-T1**	Mixed, mainly sarcomatoid	++	++	125	+	42	58	34	38	20	54
	Sarcomatoid	+	++	110	+	54	32	28	14	32	40
	Sarcomatoid	++	+	158	++	42	10	36	12	18	44
	Mixed, mainly sarcomatoid	++	+	172	++	82	30	24	20	16	10
	Sarcomatoid	+	++	146	+	62	18	40	16	24	22
	Sarcomatoid	++	++	142	+	36	12	36	18	48	24
	Sarcomatoid	++	+	160	++	96	16	58	18	18	16
	Sarcomatoid	++	+	130	++	16	20	62	22	26	32
	Sarcomatoid	++	+	108	++	86	22	48	14	22	10
	**Mean**			**139.0**		**57.3**	**24.2**	**40.6**	**19.1**	**24.0**	**26.5**

^*^For invasive tumors, the different situations analyzed corresponded to multiple metastatic localizations.

In contrast, the M5-T1 cell line was characterized by deep infiltration of abdominal organs and multiple visceral and regional nodal metastases. The atypias were marked-to-severe and the mean Ki67 index was high, between 11 to 17 per HPF. With this M5-T1 cell line, the tumor stroma was characterized by a high blood-vessel density and a low level of T-lymphocyte and macrophage infiltration, with immune cells mostly located at the periphery of the neoplastic nodules (Figure 2).

The F4-T2 and F5-T1 cell-lines showed an intermediate invasive phenotype, with neoplastic infiltration mostly located at the periphery of the omentum and viscera, but multifocal mild-to-moderate subcapsular invasion. Some intraparenchymal metastases were observed for the F5-T1 cell line. Both these cell lines were characterized by a high proliferative activity (from 14 to 24 Ki67-positive tumor cells by HPF). The blood vessel density was moderate. The stroma of these tumors was heavily infiltrated with macrophages, particularly in the center of the tumor nodules. There was also a significant infiltrate of T lymphocytes (mainly CD8 positive) both at the periphery and in the contact of neoplastic cells. This leukocytic infiltration of the stroma was more substantial with F4-T2 compared with F5-T1, both for T cells and macrophages (Figure 2).

### Characterization of the first stage: acquisition of invasive properties

**Figure 3:**
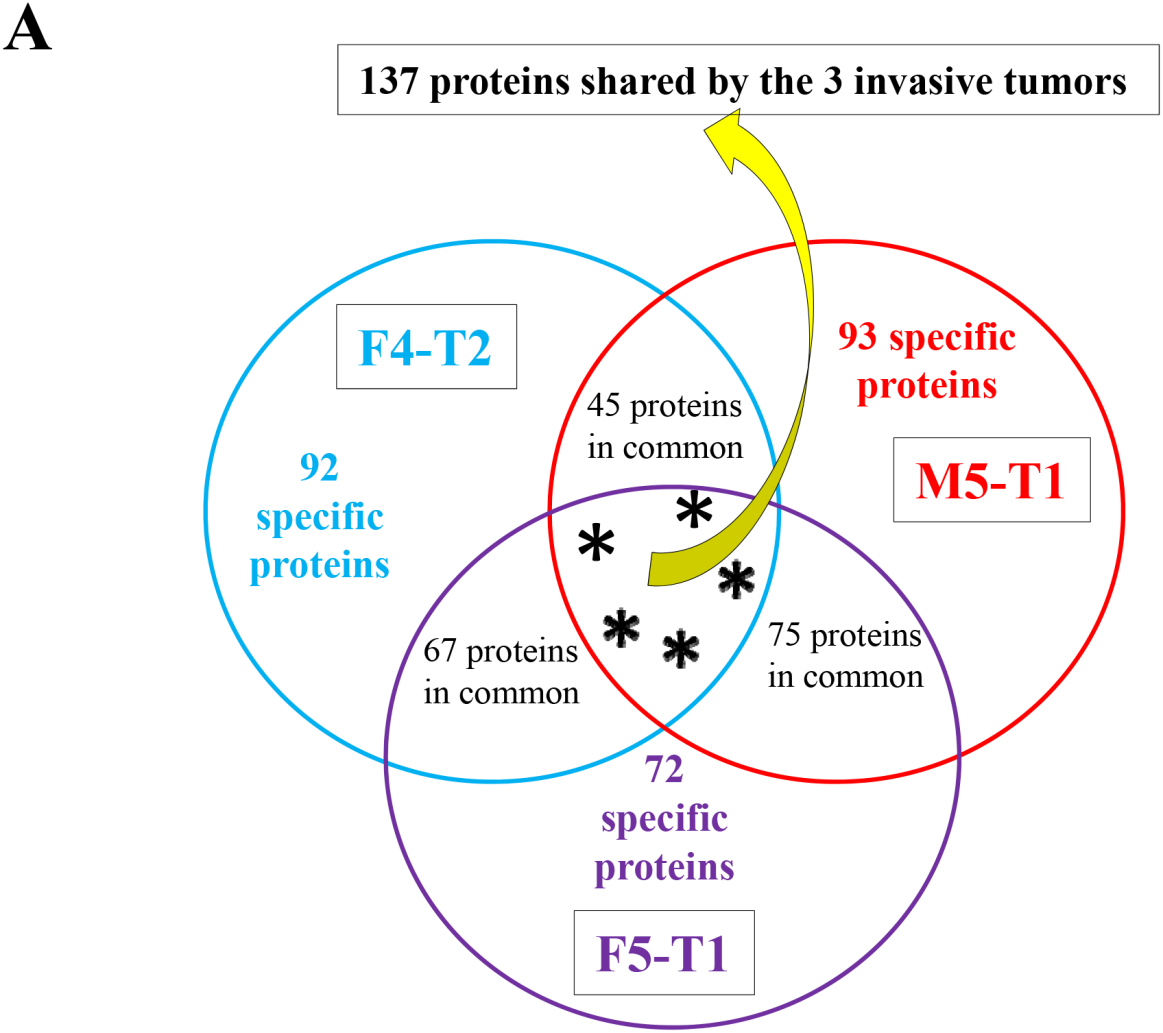

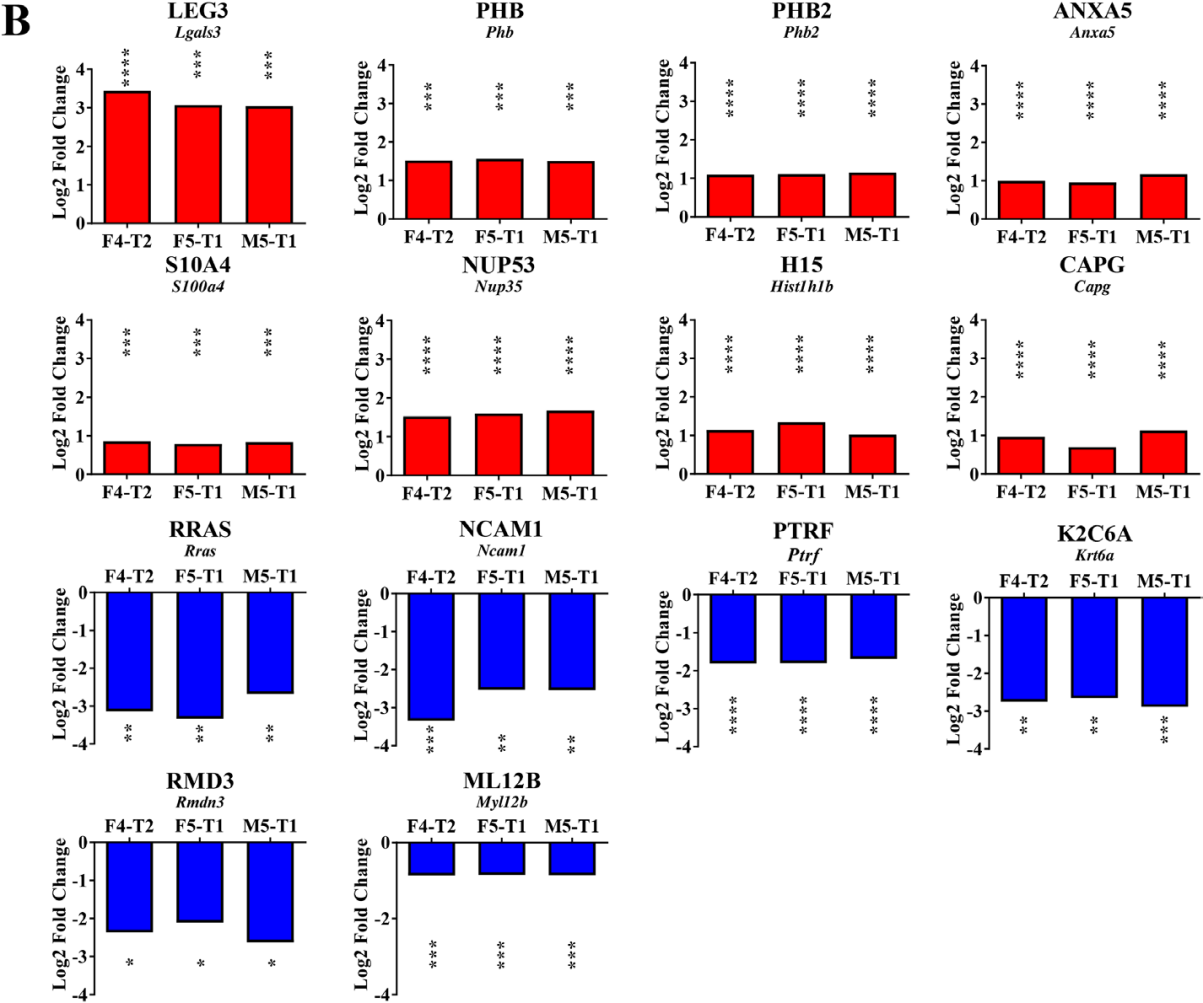
Proteins associated with the acquisition of invasive properties **(A)**, Schematic representation of the number of proteins analyzed in the comparison between the invasive tumors, F4-T2, F5-T1 and M5-T1 (M5-T2 noninvasive tumor as the reference). **(B)**, Quantitative changes of the same order of magnitude shared by the three invasive tumors (main proteins from the list of 137 indicated in (A) and described in Table S2, restricted to *p* values < 0.05 for both MSstats and MarkerView statistical analyses). Log2FC on the bar graphs correspond to the Log2 fold change observed between the three invasive tumors relative to the noninvasive tumor M5-T2. Log2FC are given for the MSstats analysis together with the corresponding *p* values (^*^0.01 < *p* < 0.05; ^**^0.001 < *p* < 0.01; ^***^0.0001 < *p* < 0.001; ^****^p < 0.0001).

**Figure 4:**
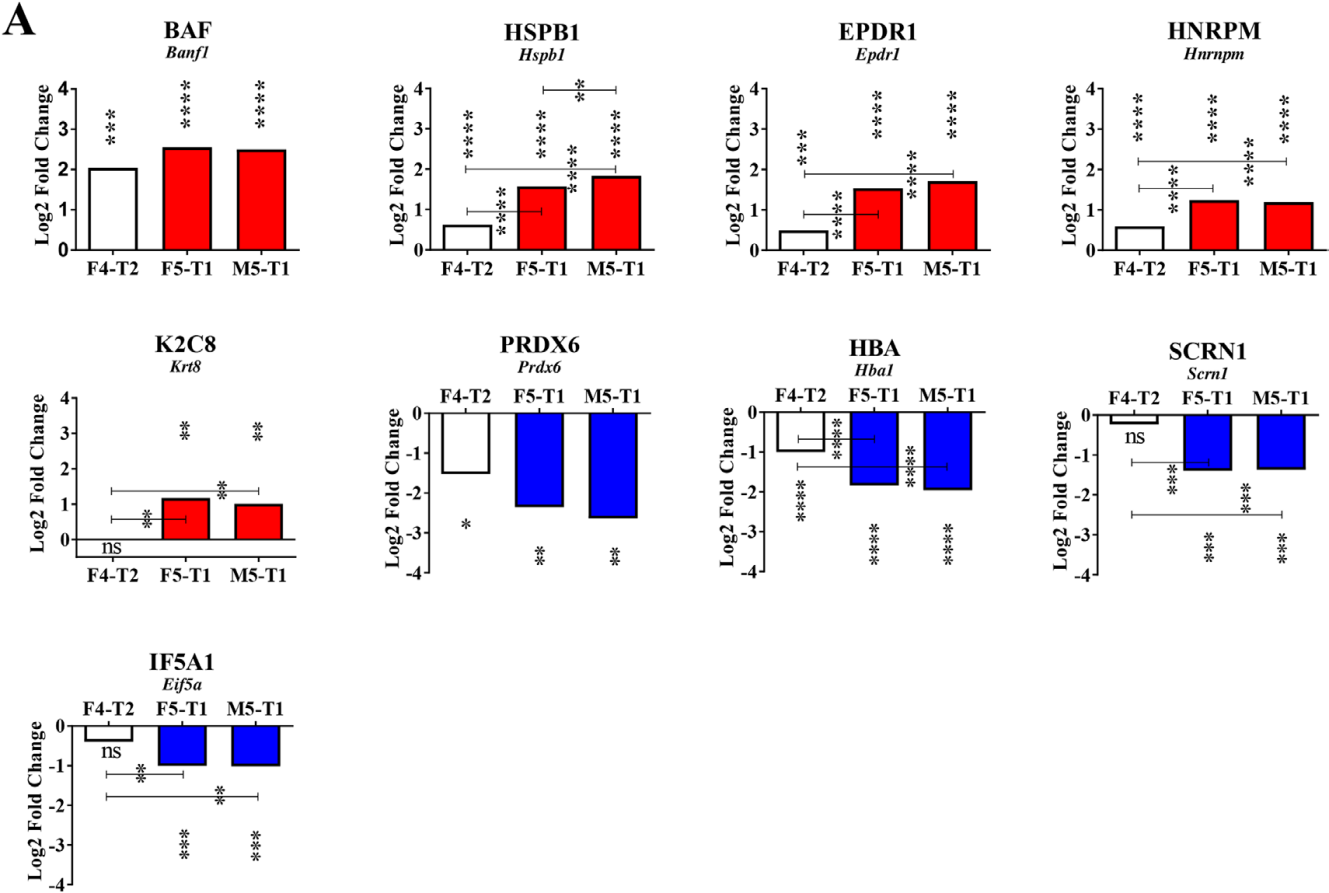

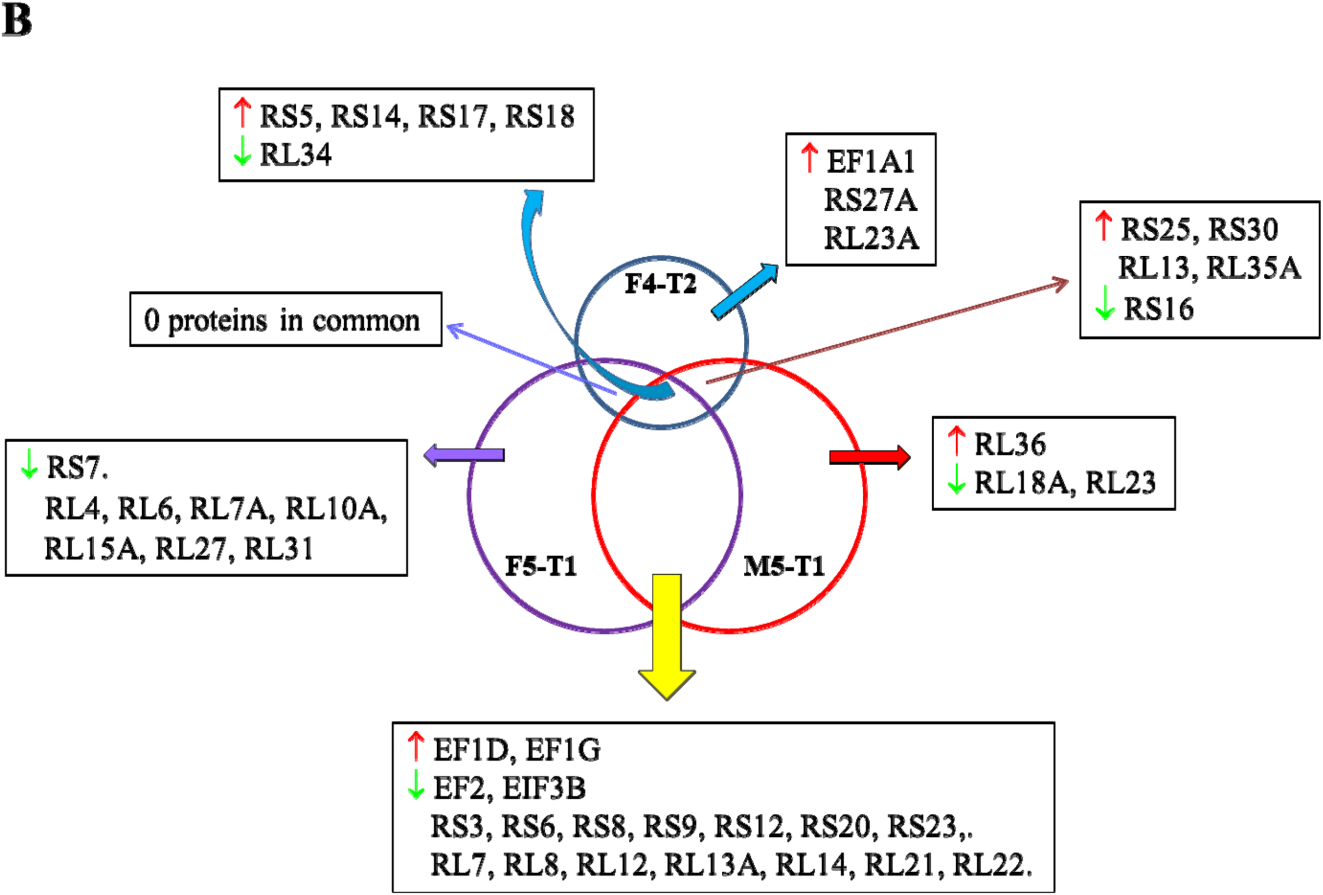
Changes associated with the increase in invasive properties **(A)**, Changes of the same order of magnitude (decrease or increase relative to the F4-T2 tumor) shared by the two most invasive tumors, F5-T1 and M5-T1. Log2FC corresponds to the Log2 fold change observed between the three invasive tumors in comparison with the noninvasive one M5-T2 (MSstats analysis and *p* values as indicated in Figure 3B). Asterisks at the top of the bars correspond to the significance of the differences observed in comparison with the noninvasive M5-T2 tumor. When significant differences were also observed between the three invasive tumors, *p* values were also indicated. **(B)**, Schematic representation of the differences and common features in the quantitative changes affecting ribosomal proteins in the three invasive tumors, F4-T2, F5-T1 and M5-T1 (M5-T2 noninvasive tumor as the reference).

**Figure 5:**
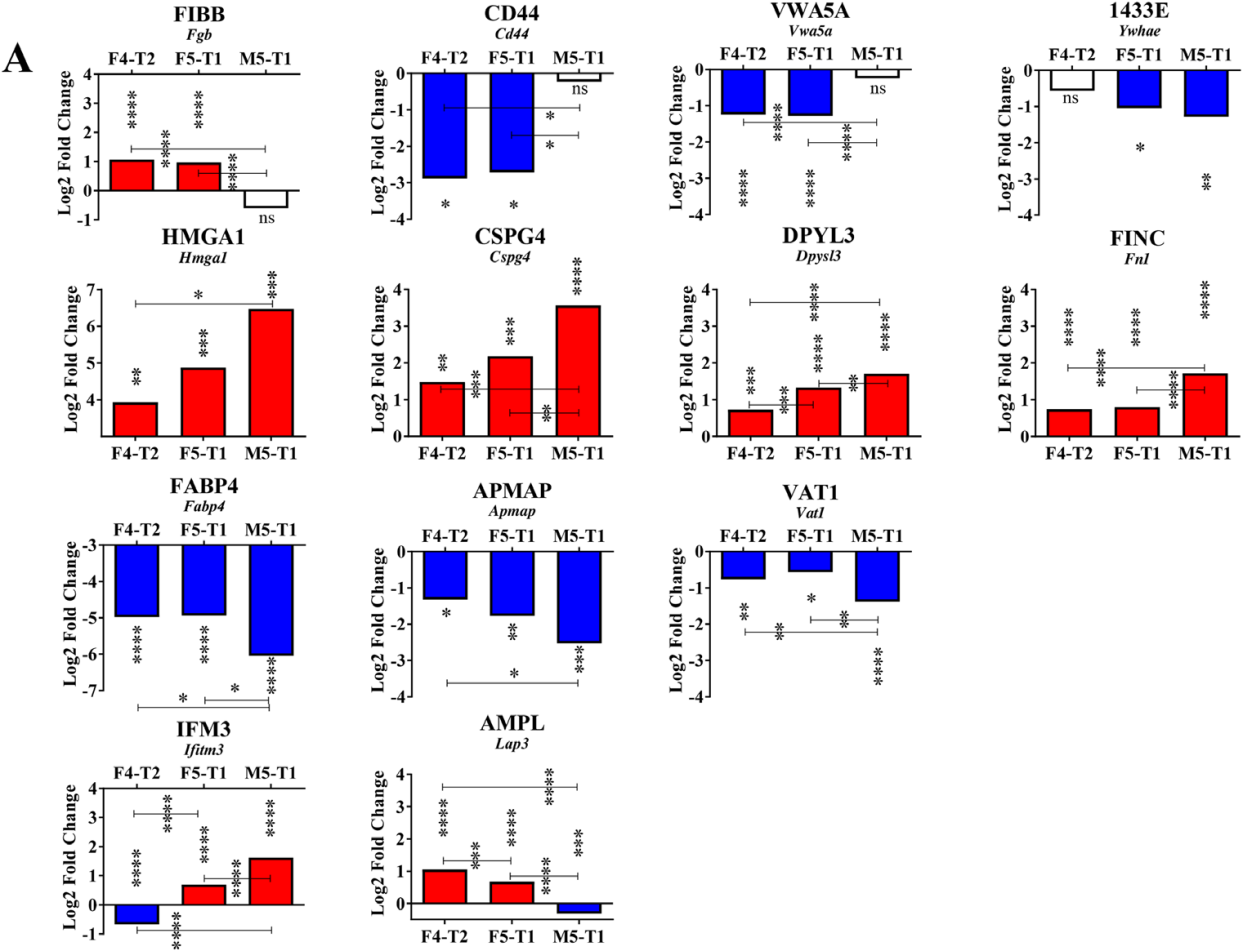

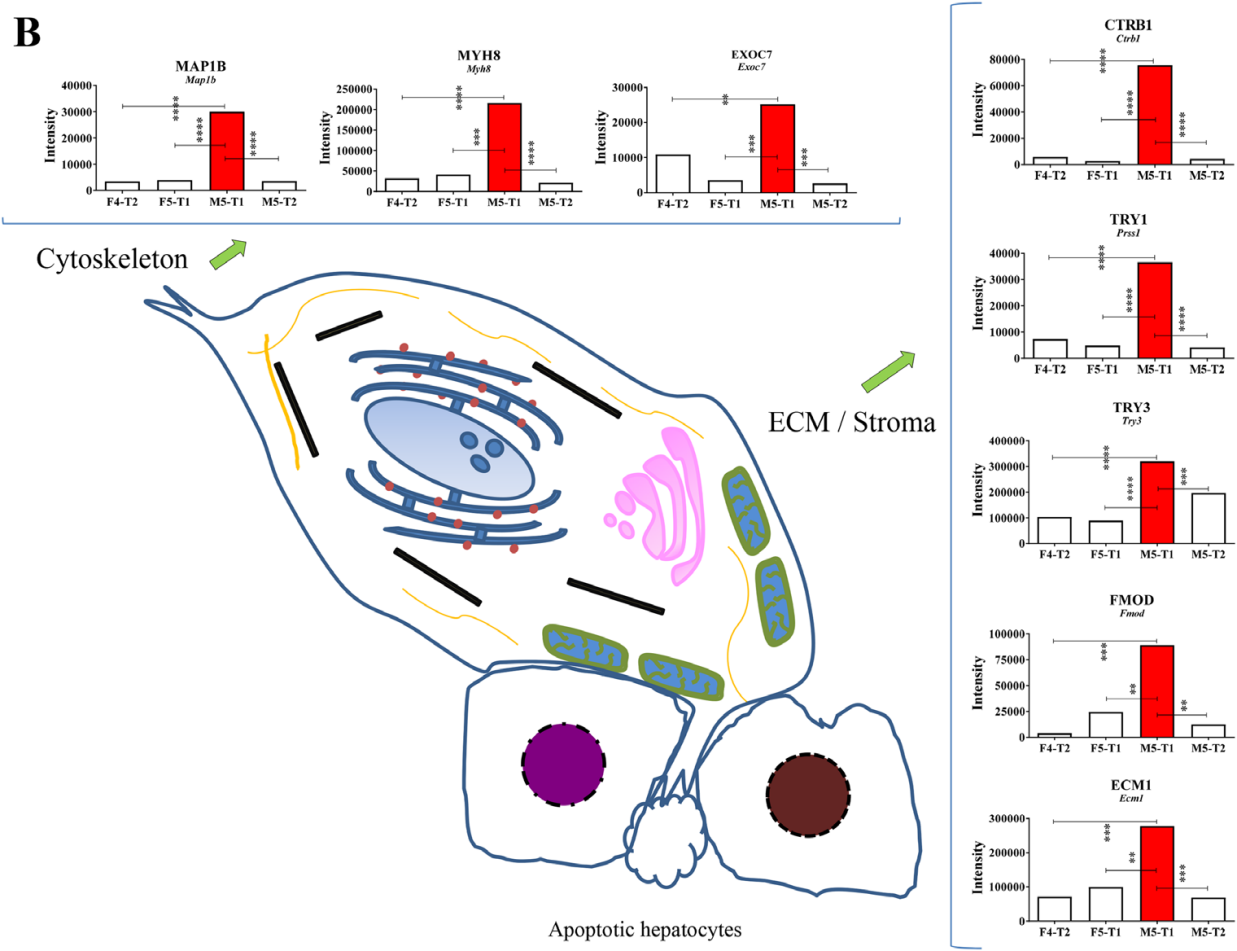
Specificities of the M5-T1 tumor **(A)**, Maximum changes (decrease or increase relative to the F4-T2 and F5-T1 tumors) observed specifically in the M5-T1 tumor. Log2FC corresponds to the Log2 fold change observed between the invasive tumors in comparison with the noninvasive tumor M5-T2 (MSstats analysis, same representation as in Figures 3B and 4A). Asterisks at the top of the bars correspond to the significance of the differences observed in comparison with the noninvasive M5-T2 tumor. When significant differences were also observed between the three invasive tumors, *p* values were also indicated. **(B)**, Schematic representation of the most aggressive M5-T1 tumor, with comparative intensities (MarkerView statistical analysis) of the proteins concerned with the most significant changes (from the list of 93 proteins exhibiting significant quantitative changes only found in the M5-T1 tumor in comparison with the noninvasive tumor M5-T2, as indicated in Figure 3A).

**Figure 6:**
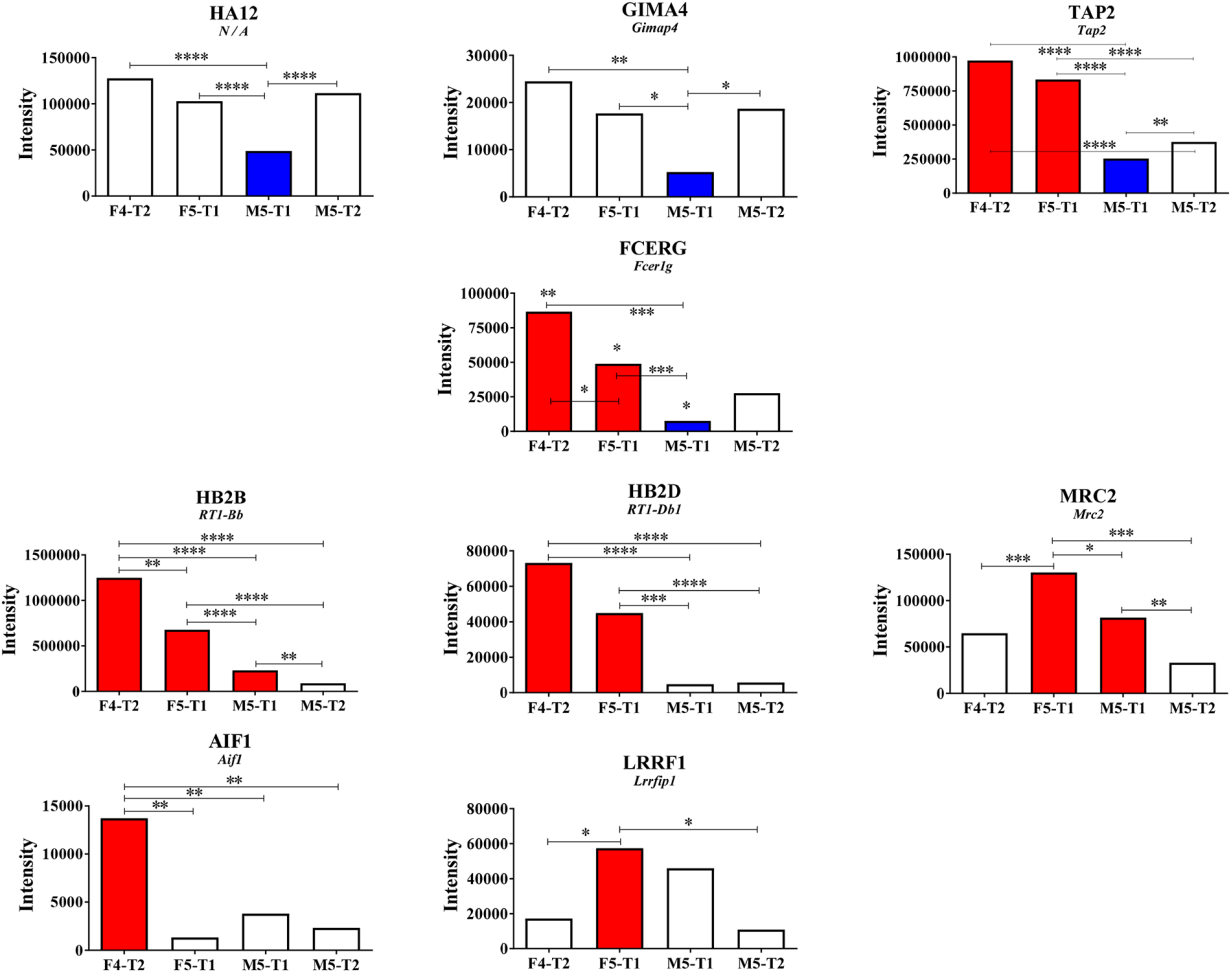
Potential immunological markers Proteins of interest related to immunological processes showing differential intensities between tumors. Asterisks correspond to the significance of the differences, when observed, in the comparisons between the four tumors (MarkerView statistical analysis).

On average, 1300 proteins were detected and identified by SWATH-MS for each tumor. SWATH-MS is characterized by a combination of optimized data dependent acquisition (DDA) for peptide identification with a data-independent acquisition (DIA) method used to extract the quantitative information of the previously identified peptides. From the DDA, a “library” containing all the information on a given identified peptide (retention time t_R_, precursor m/z and MS/MS spectra) is obtained and it is further used to extract (from the DIA file) the chromatographic elution traces of a group of specific fragment ions (peak groups) for each high confidence peptide [17]. For each listed protein, basic information on SWATH-MS acquisition was provided in Supplementary Table 1. Comparisons of each of the invasive tumors (1 = F4-T2, 2 = F5-T1, 3 = M5-T1) relative to the noninvasive tumor (4 = M5-T2) led to three lists of proteins of interest. These lists gave Log2 fold changes in protein content, for which *p* values < 0.05 were recorded for both MSstats and MarkerView statistical analysis (343 proteins for 1 vs 4, 354 proteins for 2 vs 4, and 350 proteins for 3 vs 4). Between these three lists, 137 proteins were shared by the three invasive tumors (Figure 3A and Supplementary Table 2). Figures 3B, 4A and 5A indicate the main proteins of interest identified in these lists. For the two most invasive F5-T1 and M5-T1 tumors (Figures 4A, 5A and 5B) the list of proteins of interest was finalized by consideration of some additional cases taken from the 75 proteins common to the F5-T1 and M5-T1 tumors (Figure 3A). In parallel, three complementary lists were established, corresponding to proteins for which the intensity, relative to that in the noninvasive tumor M5-T2, was found to be statistically significant only for F4-T2, F5-T1, or M5-T1. From these data, Figures 5B and 6 were generated.

A number of proteins exhibited significant increases of the same order of magnitude in the three invasive tumors relative to the noninvasive tumor, M5-T2 (Figure 3B). The most important change corresponded to galectin 3 (encoded by the *Lgals3* gene), a galactose-specific lectin associated with the cell membrane. Significantly increased fold changes also concerned probitin and prohibitin 2 (encoded by *Phb* and *Phb2* genes, respectively), annexin 5 (encoded by *Anxa5*) and protein S100A4 (protein S100-A6 (Supplementary Table 2) also showed the same pattern, data not shown). Additional proteins showing significant increases were nucleoporin (encoded by *Nup35*), a key component of the nuclear pore complex, histone H1.5 (encoded by *Hist1h1b*), a component involved in chromatin compaction (the same pattern was also observed for histone H2A.J (Supplementary Table 2), data not shown), macrophage-capping protein (encoded by *Capg*), and proteins involved in ribosomal RNA processing (data not shown).

Overall, two main categories of proteins were significantly decreased. A first category included the Ras-related protein R-ras, a member of the Ras subfamily of small G-proteins (encoded by *Rras*), neural cell adhesion molecule 1, a membrane-bound glycoprotein belonging to the immunoglobulin-like CAM family of adhesion molecules (encoded by *Ncam1*), and polymerase I and transcript release factor (encoded by *Ptrf*), a key component of the structure of caveolae, also considered as a tumor suppressor. The second category contained proteins involved in the reorganization of the actin cytoskeleton, keratin type II cytoskeletal 6A (encoded by *Krt6a*), regulator of microtubule dynamics protein (encoded by *Rmdn3*), and myosin regulatory light chain 12B (encoded by *My112b*). Five other proteins belonging to the same latter family (myosins 9, 10 and 11, myosin light polypeptide 6 and unconventional myosin 1C (Supplementary Table 2)), were also concerned with downregulation (data not shown).

### Second stage: inflammation and increased invasive properties

The F5-T1 and M5-T1 tumors shared in common an increased or decreased abundance of many proteins (relative to the F4-T2 tumor) (Figure 4A). Overall, the most significant Log2FC(in the comparison of the three invasive tumors with the noninvasive tumor, M5-T2) could be summarized in two categories, according to whether Log2FC was already significant for F4-T2, or not. In the first category the proteins concerned with the most significant changes were barrier-to-autointegration factor (encoded by *Banf1*), heat shock protein beta-1 (encoded by *Hspb1*), mammalian ependymin-related protein 1 (encoded by *Epdr1*), and heterogeneous nuclear ribonucleoprotein M (encoded by *Hnrpm*). Two proteins concerned with significantly decreased Log2FC were peroxiredoxin-6 (encoded by *Prdx6*) and hemoglobin subunit alpha-1/2 (encoded by *Hba1*). In the second category, Log2FC was increased for keratin, type II cytoskeletal 8 (encoded by Krt8) and decreased mainly for two proteins, secernin-1 (encoded by *Scrn1*), and eukaryotic translation initiation factor 5A-1 (encoded by *Eif5a*). In parallel, the F5-T1 and M5-T1 tumors were characterized by common quantitative changes affecting 18 ribosomal proteins (Figure 4B).

### Third stage: paroxysm of invasiveness, role of the extracellular matrix

The M5-T1 tumor, which presented the highest metastatic potential of the three invasive tumors, was characterized by three important observations. First, when examining Log2FC values in the comparison of the three invasive tumors with the noninvasive tumor, M5-T2, several proteins exhibited dramatic changes compared with the F4-T2 and F5-T1 tumors (Figure 5A, top row). These included: fibrinogen beta chain (encoded by *Fgb*), CD44 antigen (encoded by *Cd44*), von Willebrand factor type A domain-containing protein 5A (encoded by *Vwa5a*), and 14-3-3 protein epsilon (encoded by *Ywhae*). Secondly, among the 137 proteins all sharing significant Log2FC in the three invasive tumors (compared with the noninvasive M5-T2 tumor), 9 of these exhibited maximal or minimal values specific to the M5-T1 tumor. These 9 proteins could be classified into three different categories according to whether the Log2FC values progressively increased (Figure 5A, 2^nd^ row) or decreased in the evolution from F4-T2 to F5-T1, and to M5-T1 (Figure 5A, 3^rd^ row), or whether a different evolution was observed between the three tumors (Figure 5A, 4^th^ row). Proteins showing an increase included: high mobility group protein HMG-1/HMG-Y (encoded by *Hmga1*), chondroitin sulfate proteoglycan 4 (encoded by *Cspg4*), dihydropyrimidinase-related protein 3 (encoded by *Dpyls3*), and fibronectin (encoded by *Fn1*). Proteins with decreased content were: fatty acid-binding protein adipocyte (encoded by *Fabp4*), adipocyte plasma membrane-associated protein (encoded by *Apmap*), and synaptic vesicle membrane protein VAT-1 homolog (encoded by *Vat1*). Two proteins exhibited a different evolution of Log2FC in the three invasive tumors, interferon-induced transmembrane protein 3 (encoded by *Ifitm3*), and cytosol aminopeptidase (encoded by *Lap3*).

Thirdly, among the 93 proteins for which the intensities appeared specifically and very significantly higher than F4-T2, F5-T1 and the noninvasive M5-T2 tumor, two important categories were represented by cytoskeletal proteins and proteins of the ECM. The first category included: microtubule-associated protein 1B (encoded by *Map1b*), myosin-8 (encoded by *Myh8*), and exocyst complex component 7 (encoded by *Exoc7*). Proteins belonging to the second category were: chymotrypsinogen B (encoded by *Ctrb1*), anionin trypsin-1 (encoded by Prss1), cationic trypsin-3 (encoded by *Try3*), fibromodulin (encoded by *Fmod*), and extracellular matrix protein 1 (encoded by *Ecm1*).

### Changes in the levels of potential immunological markers

A careful analysis of all the proteins from the seven different lists, as illustrated in Figure 3A, led to the identification of ten main markers that exhibited significant quantitative changes between the four tumors and appeared to be associated with immunological processes (Figure 6). Among these, four proteins showed a downregulation specifically observed in the most aggressive M5-T1 tumor: RT1 class I histocompatibility antigen, AA alpha chain (HA12) (Günther et al. 2001), GTPase IMAP family member 4 (encoded by *Gimap4*), antigen peptide transporter 2 (encoded by *Tap2*), and high-affinity immunoglobulin epsilon receptor subunit gamma (encoded by *Fcer1g*) (Figure 6, top and 2^nd^ rows). Compared with the noninvasive M5-T2 tumor and the most invasive M5-T1 tumor, the intensity was very significantly increased in the F5-T1 and F4-T2 tumors for two proteins, B-1 beta chain and D-1 beta chain of the Rano class II histocompatibility antigen (encoded by *RT1Bb* and *RT1-Db1*, respectively, Figure 6, 3^rd^ row), an observation that was common to *Tap2* and *Fcer1g*. Three proteins appeared to be more specifically upregulated by F4-T2 or F5-T1: allograft inflammatory factor 1 (encoded by *Aif1*), leucine-rich repeat flightless-interacting protein 1 (encoded by *Lrrfip1*), and C-type mannose receptor 2 (encoded by *Mrc2*).

### Differential expression of cytokines in invasive tumors

**Figure 7:**
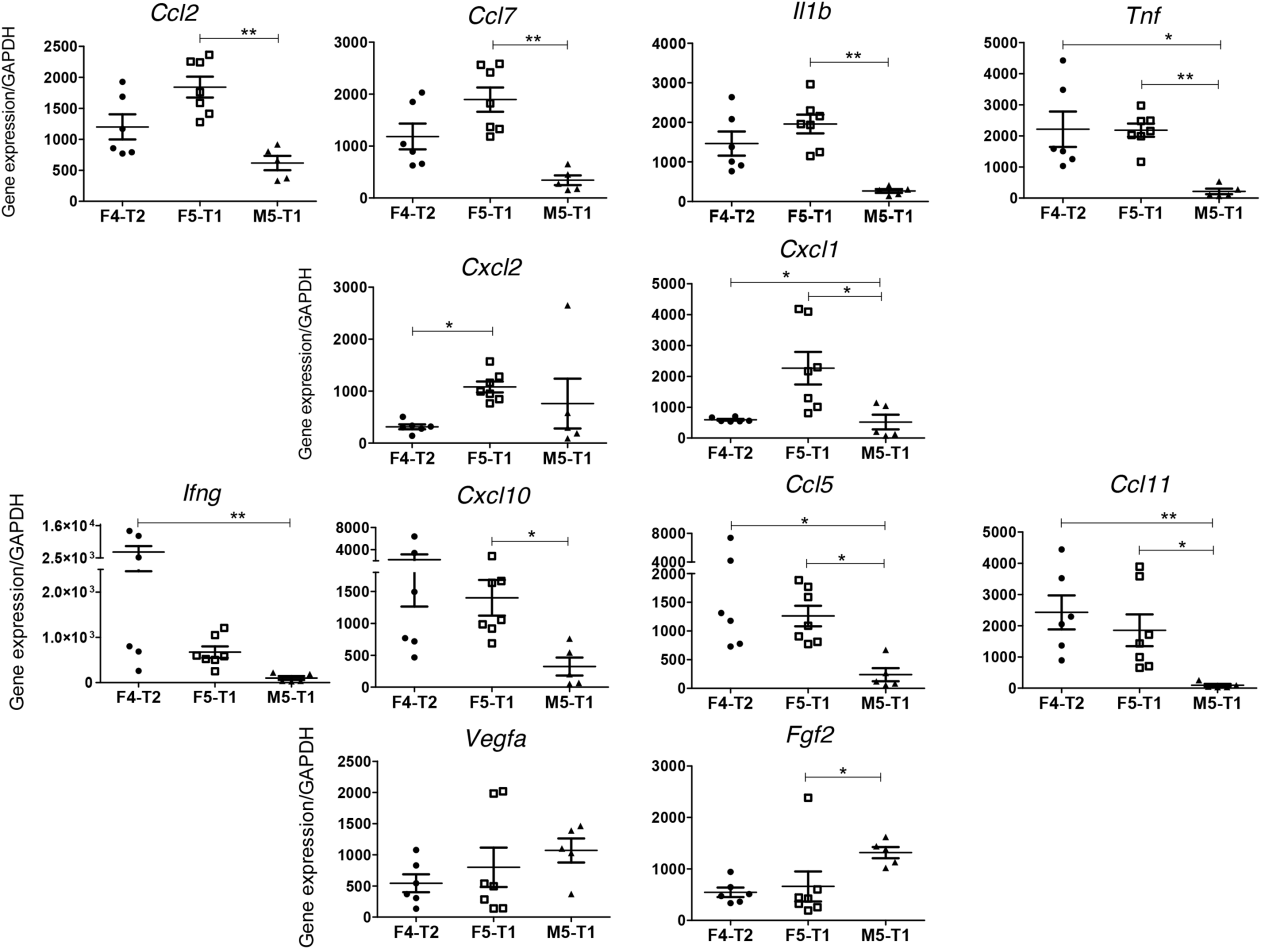
RT-PCR analysis of the expression of selected genes discriminating the three invasive tumors Top row(s), genes presenting a maximal expression in the F5-T1 tumor, related to inflammatory process and/or macrophage infiltration. Middle row, genes presenting a maximal expression in the F4-T2 tumor, related to lymphocyte infiltration. Bottom row, genes expressing a maximal expression in the M5-T1 tumor.

Analysis of the expression of a set of cytokines and growth factors by RT-qPCR led to the identification of three different profiles between the three invasive tumors. F5-T1 differed from F4-T2 and M5-T1 by the highest level of seven cytokines, suggesting a greater inflammatory reaction. Among these cytokines, the expression of *Ccl2, Ccl7, Il1b*, and *Tnf*, which represent chemoattractants for macrophages, was significantly higher than in M5-T1 (Figure 7, top row), whereas *Cxcl1* and *Cxcl2* levels were higher compared with F4-T2 (Figure 7, 2^nd^ row). Furthermore, F5-T1 and F4-T2 shared in common a significantly higher expression of *Ccl11* and *Ccl5* compared with M5-T1. Besides *Cxcl10* and *Ccl5*, which are known to be lymphocyte chemoattractants, F4-T2 was also characterized by the highest expression of *Ifng* (Figure 7, 3^rd^ row). Interestingly, M5-T1 showed the lowest expression of most cytokine genes, but was characterized by the highest expression levels of two main growth factor genes, *Vegfa* and *Fgf2* (Figure 7, bottom row).

## DISCUSSION

### Invasive properties were associated with changes in 3 main categories of proteins

Of the proteomic data, the most significant change shared in common by the three invasive MM concerned galectin-3, a multifunctional protein distributed both intracellularly and in the tumor stroma, which is involved in the development, progression, invasion and metastasis of many cancers [18]. In addition to its function in lattice formation on the cell surface, recent lines of evidence have demonstrated the role of galectin-3 in orchestrating distinct cell events in the TME [19, 20], and thus in suppressing immune surveillance [21]. The increase in nucleoporin 53 and histone H1.5 are consistent with the previous description of the involvement of four other nuclear pore proteins [22] in cancer, and with the strong positive staining of H1.5 in different tumors types compared to the homologous normal tissue, particularly those of higher grade [23]. The increase in four multifunctional proteins, prohibitin and prohibitin 2 [24], annexin A5 [25] and protein S100A4 [26], has also been documented in cancers, contributing to tumorigenic processes such as cell proliferation, metastasis, angiogenesis and immune evasion.

In our current study, the decreased content observed in invasive tumors could also be explained by the ability of RRAS to stimulate cell adhesion, in addition to many other functions [27]. NCAM belongs to the same category of proteins involved in cell adhesion, as both upregulation and downregulation have been reported in cancers, however inactivation of the *Ncam* gene *in vivo* has indicated a key role for this adhesion molecule in governing the interplay between tumor cells and their microenvironment [28]. At the membrane level, PTRF is also described as a key component of caveolar structure, for which downregulation correlates with advanced stages of colorectal cancers [29]. Another category of proteins involved in the shaping of the cytoskeleton includes CAPG, for which the increased content is in accordance with its involvement in migration and invasiveness [30]. The decrease in ML12B and other myosins found in the three invasive tumors may be related to the fact that myosin regulatory light chains are essential for cellular integrity [31]. Regarding K2C6A, the last decade has provided evidence for active keratin involvement in cancer cell invasion and metastasis [32], loss of cytokeratin expression being associated with more-aggressive tumors [33], particularly during the EMT of cancer cells. This observation is corroborated by our previous findings on the most aggressive MM cell line, M5-T1, as immunofluorescence staining of cell monolayers revealed that only a few cells retained this epithelial differentiation marker [34]. Even more strikingly, the case of RMD3 retains much attention, as this regulator of microtubule dynamics is involved in the formation of tight functional contacts between the endoplasmic reticulum (ER) and mitochondria [35], suggesting that perturbations in ER–mitochondria signaling have a profound impact on the acquisition of invasive properties.

### Higher metastatic potential was associated with changes in additional proteins

In this study, several lines of evidence demonstrate that F5-T1 and M5-T1 tumors present a higher degree of invasiveness relative to F4-T2. Among the proteins concerned by the most significant positive Log2FC values shared by F5-T1 and M5-T1, two nuclear proteins appear: BAF and HNRPM. The barrier-to-autointegration factor (BAF) plays a crucial role in chromatin organization, and its frequent overexpression in cancers now represents a target for therapy [36, 37]. HNRPM is a member of the family of heterogeneous nuclear ribonucleoproteins, which was initially described as a regulator of splicing [38]. However, the role of a receptor that this protein plays with the carcinoembryonic antigen reveals that it is a mediator of metastasis and inflammatory response [39]. In parallel, many ribosomal proteins were affected in the comparison of F4-T2 with F5-T1 and M5-T1, emphasizing their roles in tumorigenesis and immune signaling in addition to their essential housekeeping functions in ribosome biogenesis and protein production [40]. Finally, a growing interest has emerged recently in the dysregulation of translation initiation factors in cancer, a common feature of tumorigenesis. Our observation of a decreased abundance of IF5A1 agrees with previous findings observed in various types of cancers [41].

Regarding the detoxifying enzyme peroxiredoxin 6, its new reduction in the F5-T1 and M5-T1 tumors tends to confirm that, as this protein represents the most prominent thiol peroxidase of the cell, capturing nearly all the H_2_O_2_ generated [42], H_2_O_2_ signaling is a major second messenger associated with invasiveness [43]. In association with oxidative stress, the decreased abundance of hemoglobin subunit α-1/2 suggests that the two more invasive tumor types exhibit a much higher level of hypoxia compared with F4-T2. HSPB1 – a member of the small heat shock proteins for which overexpression in many cancer cells has been associated with an anti-apoptotic role – also presents links with cytoskeletal components [44], the marked increased content in HSPB1 in the F5-T1 and M5-T1 tumors being correlated with an increase in the abundance of K2C8 [32]. The decrease in secernin 1, involved in the regulation of intracellular trafficking within the cell is also consistent with the downregulation of secernin 1 that was previously observed in prostate cancer in comparison with normal prostatic tissue [45]. Finally, the rise in ependymin content raises the interesting question of its anti-adhesive properties [46].

### Highest invasiveness was characterized by specific immune-proteomic-cytokine profiles

Overall, our results tend to demonstrate that the most aggressive of the three invasive MM was M5-T1. This tumor cell line showed the highest invasive capacity *in vitro* [34] while deep infiltration of abdominal organs and intraparenchymal and nodal metastases were observed *in vivo*. Important significant changes in protein levels were, specifically, observed with this tumor. At the nuclear level, M5-T1 was characterized by a maximal Log2FC increase in HMGA1, which suggests an involvement in nucleotide excision repair [47]. Secondly, two proteins connected to lipid metabolism were also concerned, suggesting a probable link with dysregulated adipose endocrine function [48]. The first one, FABP4, which belongs to a family of intracellular lipid chaperones coordinating the distribution and function of lipids within cells, has been identified as a protective factor to strengthen IFN responses against tumor growth [49]. Interestingly, as for another protein of this category, APMAP, our data show that IFN-γ expression was minimal in this tumor. This emphasizes the existence of an important link with prohibitin expression and monocytic macrophages and dendritic cells, as this protein is selectively expressed in these cells [50], but also with the strong immunosuppressive properties of this tumor attested by the low number of infiltrating T-cells and macrophages, and the minimal expression of CXCL10, CCL5, and CCL11. This differential behavior of M5-T1 compared with F4-T2 and F5-T1 was also observed for FIBB, CD44, and VWA5A, in agreement with the fact that both CD44 and VWA5A were interesting markers for detection of the early stages of colon cancer in the plasma [51]. Finally, an intriguing feature was the new decrease in VAT1, which, in light of the recent work of Mendes et al., raises the question of a probable deregulation of exosome secretion [52].

In correlation with the low numbers of CD8+ T-cells observed in the tumor stroma, evidence of the immunosuppressive character of the M5-T1 tumor was provided by the downregulation of two proteins, a member of the RT1 class I histocompatibility complex of the rat [53], a crucial element for the induction of CD8+ T-cell adaptive immune responses against tumors [54], and GIMA4, which belongs to the immunity-associated protein family involved in T-lymphocyte biology [55], two members of this family being downregulated in hepatocellular carcinoma compared with normal tissues [56]. Associated with the decrease in GIMA4 and HA12, our study demonstrated that the abundance of cytosol aminopeptidase (AMPL), a protein that influences MHC class I-mediated antigen presentation [57], was affected in M5-T1 in comparison with the F4-T2 and F5-T1 tumors. A decrease in TAP2, involved in peptide transport from the cytosol into the endoplasmic reticulum [58], was also observed specifically in the M5-T1 tumor. Finally, another dramatic decrease concerned FCERG, which regulates several aspects of the immune response [59]. All these findings lead to the suggestion that in this tumor several aspects of the dynamic process of MHC class I antigen presentation are not functional [57, 60], leading to tumor escape from immune recognition.

### Highest invasiveness was associated with specific changes in ECM and cytoskeletal proteins

The M5-T1 tumor was also specifically characterized by important changes in the content of several transmembrane proteins and components of the extracellular matrix. Among proteins belonging to the first category, IFM3 was reported to be highly expressed in invasive phenotypes of different types of cancer [61, 62]. CSPG4, a cell surface proteoglycan, was overexpressed in a huge range of human and animal tumors, the tumor microenvironment, and cancer initiating cells [63]. The overexpression of DPYL3, a member of the dihydropyrimidinase-related proteins, a family of membrane-associated proteins involved in microtubule assembly, was first reported to be related to cancer three years ago [64]. In this context, the dramatic increase in fibronectin content, which was consistent with the specific maximal expression in FGF2 of this tumor, suggests that fibronectin plays an essential role as a critical mechanoregulator of the ECM [65]. Interestingly, another specificity of the M5-T1 tumor was an increased abundance of other proteins involved in the reorganization of the ECM, such as: chymotrypsinogen B and trypsins, which have been found to be elevated in tissue interstitial fluids of colorectal cancer [66]; fibromodulin, which was revealed as an important regulator of glioma cell migration [67]; and extracellular matrix protein 1, for which overexpression is related to a very poor diagnosis in various cancers [68, 69]. Another set of events also occurred at the level of cytoskeletal proteins, which could explain the most invasive potential acquired by M5-T1 tumor cells. Among these proteins, MAP1B, is a key actor of microtubule stability linked to the dynamic rearrangements required for malignant cells to move and metastasize [70]. EXOC7 is another protein representing a link between ECM degradation, cytoskeleton, and acquisition of the highest metastatic capacity, as a member of the exocyst participating in the formation of invadopodia, which are key secretion sites for exosomes [71]. Yau et al. demonstrated that the gene coding for this protein belonged to a set of 14 gene candidates that were outcome predictors of distant metastatic relapses in a cohort of patients with triple-negative breast cancers [72]. Finally, another member of the family of non-muscle myosins (mentioned above), myosin-8, is an actin-dependent molecular motors, which enhanced expression is involved in the regulation of cancer cell migration and invasion [73].

### Differential immune-proteomic-cytokine profiles between F5-T1 and F4-T2 tumors

In contrast to M5-T1, the F4-T2 and F5-T1 tumors shared in common higher expression levels of some cytokine genes (*Ccl2, Ccl5, Ccl7, Ccl11, Tnf*) and higher contents of TAP2, FCERG, and MHC class II antigens. These data are consistent with histological and immunohistochemical observations, showing an important infiltration of these tumors with immune cells, including CD3+ T-cells and macrophages. However, these two tumors differed on many points. The higher level of FCERG and the MHC class II antigens, HB2B and HB2D, in the F4-T2 tumor suggests a more important immunoreactivity [74], corroborated by a higher mean *Ifng* expression and a higher immune cells infiltration in the tumor stroma on histoslides. The higher mean expression level of *Cxcl10* relative to that of *Ccl2* or *Ccl7* in the F4-T2 tumor in comparison with F5-T1 agrees with our analyses of the number of CD8+ versus ED1+ cells infiltrated in the two tumors, and with previous reports that *Cxcl10* expression in the TME is associated with a higher level of T-cell infiltration [75]. Finally, the more substantial increase in *Ccl11* expression in F4-T2 than in F5-T1, compared with M5-T1, could also reflect a greater recruitment of leukocytes, in agreement with findings by Wågsäker et al. [76].

Conversely, the lower *Ccl5* to *Ccl7* and *Ccl5* to *Ccl2* expression ratios in F5-T1 support a TME containing less T-lymphocyte chemoattractants [77] and with a greater inflammatory reaction, also attested by the maximal fibrinogen content that characterized this tumor. Two other cytokine gene expressions evolved in the same way, *Cxcl1* and *Cxcl2*, suggesting that these chemotactic factors for neutrophil recruitment are produced by TAMs and/or tumor cells in response to inflammatory stimuli [78, 79]. Among the proteins showing, specifically, a greater content in the F5-T1 tumor, the ITIH3 protein also appears to be related to the function of macrophages in inflammation, in agreement with reports from Bourguignon et al. [80] and Gomez-Toledo et al. [81]. The specific high content of AIF1 in the F4-T2 tumor versus F5-T1 may also be related to a higher immune cell infiltration [82]. Finally, besides these features, the fact that the content of LRRFIP1 and MRC2 was substantially higher in F5-T1 compared with F4-T2 could be explained by a greater inflammatory infiltrate of F5-T1 [83, 84].

In conclusion, the specific cellular and molecular features presented by our four experimental rat MM models represent interesting new tools for basic research in oncoimmunology and oncoproteomics on the most aggressive types of cancers. The different proteins concerned by quantitative changes, which characterized each of the three invasive stages identified in our study, raise several fundamental questions of interest for future investigations on the connections between different signaling pathways within cancer cells, and on stromal/cancer cell crosstalk within the tumor tissue. Another interesting prospect could be the validation of the set of potential markers in human specimens. Finally, these four models might represent a good basis for the evaluation of innovative therapies, alone or combined, in particular in the field of immunotherapy.

## MATERIALS AND METHODS

### Cell culture

The M5-T2, F4-T2, F5-T1 and M5-T1 cell lines used in this study belong to a biocollection of 27 preneoplastic and neoplastic F344 rat cell lines (https://migratech.inserm-transfert.fr/srv/tech/2/index100.asp?cl=CL) established in 2011, which was characterized in a previous study [15]. All the cells were maintained in RPMI-1640 medium supplemented with 10% heat-inactivated fetal calf serum, 100 U/mL penicillin, 100 μg/mL streptomycin and 2 mM L-glutamine (all reagents from Gibco-Invitrogen) and cultured at 37°C in a 5% CO_2_ atmosphere.

### Generation of intraperitoneal (IP) syngeneic models of MM in immunocompetent F344 fischer rats

Fischer F344 rats were obtained from Charles River Laboratories (L’Arbresle, 69, France) and maintained under standard conditions, according to institutional and European Union guidelines for the care and use of laboratory animals in research protocols (Agreement # 01257.03, French MESR Ministry and Regional ethics committee of the Pays de la Loire, France). Rats were fed a pelleted standard diet (RM1, Special Diet Services, Witham, Essex, UK), with tap water *ad libitum*, and were anesthetized via an isoflurane chamber (Forene^®^, Abbott France) and euthanized with Dolethal^®^ (Centravet, Pluduno, Plancoët, France).

Orthotopic injections of M5-T2, F4-T2 (5 x 10^6^ cells in 300 μl RPMI-1640 unsupplemented medium), F5-T1 or M5-T1 cells (3 x 10^6^ cells in 200 μl RPMI-1640 unsupplemented medium) were performed into the peritoneal cavity of 12 week-old male Fisher rats. M5-T1- and F5-T1-treated rats bearing tumors were euthanized 2.5 and 3.5 weeks post MM cell injection, respectively. F4-T2- and M5-T2-treated rats bearing tumors were euthanized 4 and 5 weeks post MM cell injection, respectively. Following euthanasia, all tissues with small metastatic nodules or residual tumor tissue were collected and either fixed in 4% paraformaldehyde (Electron Microscopy Sciences, Hatfield, USA) or frozen at −80°C for further analysis.

### Histology and immunohistochemistry

For histological examination, the paraformaldehyde fixed, paraffin-embedded sections of rat tumor samples and surrounding invaded tissues, when present, were cut with a Bond Max automaton (Menarini, Rungis, France) and stained with hematoxylin-phloxine-saffron (HPS). Antibodies used for immunohistochemical analyses were: anti-human Ki67 (rabbit monoclonal SP6 clone 1/100, Spring Bioscience, Pleasanton, CA, USA) and anti-human von Willebrand Factor (rabbit polyclonal 1/400, Agilent Technologies, Les Ulis, France) with the IView Universal DAB detection kit (Roche Diagnostics) for revelation; and anti-rat ED1 antibody (MAB1435 1/100, EMD Millipore Corporation, Billerica, MA, USA) used as a pan-macrophage marker, mouse anti-CD3 (SM253P, Acris Antibodies, San Diego, USA), anti-CD8 (LS-B3665, LSBio France, 92000 Nanterre), with an anti-mouse secondary antibody and N-Histofine Simple Stain Mouse MAX Peroxidase (Nichirei Biosciences, Tokyo, Japan) as the detection reagent. Histopathology slides were scanned with a Nanozoomer 2.0 HT (Hamamatsu Photonics K. K., Japan) and photographs of slides were taken using an Eclipse 50i microscope and a Nikon DS Fi-1 digital camera (Nikon Instruments Europe B.V.) Semiquantitative evaluation nand scoring of the immunohistochemical staining were performed blindly by a pathologist. Ki-67, CD3, CD8 and ED1 were assessed based on the number of positive cells counted on 10 High Power Fielf (HPF) by manula image analysis involving the use of the image J software, Research Servic Branch, National Institute of Health, Bethesda, Maryland, USA). Negative controls for IHC were included in each run, and consisted in replacing the primary antibody with normal mouse or rabbit serum (prediluted reagents, Roche Diagnostics).

### SWATH-MS analysis

### Creation of the spectral library

To create a spectral library, we used three types of samples from rat cell lines, frozen normal tissues and formalin-fixed paraffin-embedded tissue sections of the four tumor models [15], and we performed DDA experiments. Each sample (5 μg) was separated into a nano 2D-LC 425 system (Eksigent) using a chromxp C18CL column (3 μm, 120 A, 15 x 0.3 cm, Sciex) at a flow rate of 5 μL/min. Water and ACN, both containing 0.1% formic acid, were used as solvents A and B, respectively. The following gradient of solvent B was used: 0 to 5 min 5% B, 5 to 75 min 5% to 35% B, then 10 min at 95% B, and finally 10 min at 5% B for column equilibration. As the peptides eluted, they were directly injected into a hybrid quadrupole-TOF mass spectrometer Triple TOF 5600 + (Sciex, Redwood City, CA, USA) operated with a ‘top 30’ data-dependent acquisition system using positive ion mode. The acquisition mode consisted of a 250 ms survey MS scan from 400 to 1250 m/z, followed by an MS/MS scan from 230 to 1500 m/z (75 ms acquisition time, 350 mDa mass tolerance, rolling collision energy) of the top 30 precursor ions from the survey scan.

Peptide identification and library generation were performed with Protein Pilot software (v4.5, Sciex^®^) using the following parameters: (1) search against a database composed by Rattus Norvegicus from SwissProt (release at February 2016, with 20254 entries), and iRT peptide sequences, and using (2) MMTS as fixed modification; (3) trypsin digestion (with a miss cleavage factor of 0.75, Paragon™ Algorithm). An independent False Discovery Rate (FDR) analysis using the target-decoy approach provided by Protein Pilot™ was used to assess the quality of identifications. Positive identifications were considered when identified proteins and peptides reached a 5% local FDR. A specific library of precursor masses and fragment ions was created by combining all files from the DDA experiments

### Relative quantification by SWATH acquisition

Each sample (5 mg) was analyzed using the LC–MS equipment and LC gradient described above for building the spectral library, but using a SWATH-MS acquisition method. The method consisted of repeating the whole gradient cycle, which consisted of the acquisition of 32 TOF MS/MS scans of overlapping sequential precursor isolation windows (25 m/z isolation width, 1 m/z overlap, high sensitivity mode) covering the 400 to 1200 m/z mass range, with a previous MS scan for each cycle. The accumulation time was 50 ms for the MS scan (from 400 to 1200 m/z) and 100 ms for the product ion scan (230 to 1500 m/z), thus making a 3.5 s total cycle time.

### SWATH MS data extraction and statistical analysis

Peak extraction of the SWATH data was performed using either the Spectronaut software (ver 8.0, Biognosys, Switzerland) or SWATH micro App embedded in PeakView (ver2.0, Sciex). SWATH data were processed with default settings in Spectronaut. Reference peptides from the iRT-kit (Biognosys) spiked into each sample were used to calibrate the retention time of extracted peptide peaks using Spectronaut. Peptide identification results were filtered with a q-value of < 1%, and excluding shared peptides. RT calibration was also performed based on iRT peptide elution profiles in PeakView using the SWATH App module (v2.0). After peak extraction with either Spectronaut or PeakView, the sum of MS2 ion peak areas of SWATH quantified peptides for individual proteins were exported to calculate the protein peak areas. For statistical analysis of the SWATH data set, peak extraction output data matrix from PeakView was imported into MarkerView (v2, Sciex) and MSstats (R package, Bioconductor) for data normalization and relative protein quantification. Proteins with a fold change > 1.5 and statistical p-value < 0.05 estimated by MarkerView and MSstats were regarded differentially expressed under different conditions.

### Total RNA isolation and real-time PCR

Tumor tissues were disrupted using an MP FastPrep-24 Instrument (MP Biomedicals Inc.). Total RNA was extracted using the NucleoSpin RNA II Kit according to the manufacturer’s instructions (Macherey-Nagel, Hoerdt, France). The total RNAs were next treated with an rDNase solution to remove contaminating genomic DNA, and subsequently purified. Total RNA (0.5 μg) was reverse transcribed using MMLV reverse transcriptase (Invitrogen). PCR reactions were conducted using QuantiTect primer assays (Qiagen) and Maxima SYBR Green/ROX qPCR Master Mix (Fisher Scientific) according to the manufacturer’s instructions. Gene expression was analyzed in non-infected and infected cells using QuantiTect primers pairs for *Ccl2, Il1b, Tnf, Cxcl2, Cxcl1, Ccl7, Ifng, Cxcl10, Ccl11, Ccl5, Vegfa, and Fgf2*. The gene expression was expressed as relative expression compared to the expression of the housekeeping gene glyceraldehyde-3-phosphate dehydrogenase (GAPDH).

### Statistical analysis

Statistical analysis was performed using GraphPad Prism 6.00 software (GraphPad Software Inc.). For statistical analysis comparing more than two groups, nonparametric one-way ANOVA (Kruskal-Wallis test) was used, with Dunn’s post test. All data are presented as mean ± SEM. P values less than 0.05 were considered to be statistically significant. ^*^p < 0.05, ^**^p < 0.01, ^***^p < 0.001.

## SUPPLEMENTARY MATERIALS TABLES






