# Supplementary material for: Characterization of increasing stages of invasiveness identifies stromal/cancer cell crosstalk in rat models of mesothelioma
Source: Oncotarget. 2018 Mar 27;9(23):16311–29. doi: 10.18632/oncotarget.24632 (PMC5893242; doi:10.18632/oncotarget.24632)
Supplement: Supplementary file 1 [file oncotarget-09-16311-s001.pdf]

Characterization of increasing stages of invasiveness identifies stromal/cancer cell crosstalk in rat models of mesothelioma

SUPPLEMENTARY MATERIALS

Supplementary Table 1: Identification of SWATH-MS. For each protein (column F), the list of peptides is given in column B, and their respective m/z and retention time values are provided in columns C and D, respectively

See Supplementary File 1

Supplementary Table 2: Complete list of the 137 proteins presenting significant quantitative changes shared in common by the three invasive tumors (relative to the noninvasive M5-T2 tumor). The list is restricted to proteins for which p values < 0.05 were recorded for both MSstats and MarkerView statistical analysis

See Supplementary File 2
