# Supplementary material for: Characterization of increasing stages of invasiveness identifies stromal/cancer cell crosstalk in rat models of mesothelioma
Source: Oncotarget. 2018 Mar 27;9(23):16311–29. doi: 10.18632/oncotarget.24632 (PMC5893242; doi:10.18632/oncotarget.24632)
Supplement: Supplementary file 3 [file oncotarget-09-16311-s003.docx]

**Supplemental Table S2**: list of proteins sharing significant Log2FC (compared with the non invasive M5-T2 tumor) common to the three invasive tumors F4-T2, F5-T1 and M5-T1.

| **Code** | **Protein name** |
| --- | --- |
| 1433F | 14-3-3 protein eta |
| AASD1 | Alanyl-tRNA editing protein Aarsd1 |
| ACADL | Long-chain specific acyl-CoA dehydrogenase, mitochondrial |
| ACSL1 | Long-chain-fatty-acid—CoA ligase 1 |
| ACTN1 | Alpha-actinin-1 |
| AKAP2 | A-kinase anchor protein 2 |
| AL1A3 | Aldehyde dehydrogenase family 1 member A3 |
| ALBU | Serum albumin |
| AL7A1 | Alpha-aminoadipic semialdehyde dehydrogenase |
| AMPL | Cytosol aminopeptidase |
| ANM1 | Protein arginine N-methyltransferase 1 |
| ANXA2 | Annexin A2 |
| ANXA4 | Annexin A4 |
| ANXA5 | Annexin A5 |
| ANXA8 | Annexin A8 |
| AOC3 | Membrane primary amine oxidase |
| APMAP | Adipocyte plasma membrane-associated protein |
| ARFG3 | ADP-ribosylation factor GTPase-activating protein 3 |
| BAF | Barrier-to-autointegration factor |
| BCAT2 | Branched-chain-amino-acid aminotransferase, mitochondrial |
| CAH3 | Carbonic anhydrase 3 |
| CALM | Calmodulin |
| CAPG | Macrophage-capping protein |
| CBPQ | Carboxypeptidase Q |
| CO1A2 | Collagen alpha-2(I) chain |
| CO4 | Complement C4 |
| COR1A | Coronin-1A |
| COX2 | Cytochrome c oxidase subunit 2 |
| COX5B | Cytochrome c oxidase subunit 5B, mitochondrial |
| CRYAB | Alpha-crystallin B chain |
| CSPG4 | Chondroitin sulfate proteoglycan 4 |
| DAPK3 | Death-associated protein kinase 3 |
| DCPS | M7GpppX diphosphatase |
| DDAH1 | N(G), N(G)-dimethylarginine dimethylaminohydrolase 1 |
| DKC1 | H/ACA ribonucleoprotein complex subunit 4 |
| DPEP1 | Dipeptidase 1 |
| DPYL3 | Dihydropyrimidinase-related protein 3 |
| ECHD1 | Ethylmalonyl-CoA decarboxylase |
| EFTU | Elongation factor Tu, mitochondrial |
| EHD2 | EH domain-containing protein 2 |
| ENOA | Alpha-enolase |
| EPDR1 | Mammalian ependymin-related protein 1 |
| EZRI | Ezrin |
| FABP4 | Fatty acid-binding protein, adipocyte |
| FAS | Fatty acid synthase |
| FCERG | High affinity immunoglobulin epsilon receptor subunit gamma |
| FIBG | Fibrinogen gamma chain |
| FINC | Fibronectin |
| FKB1A | Peptidyl-prolyl cis-trans isomerase FKBP1A |
| FUBP2 | Far upstream element-binding protein 2 |
| G3P | Glyceraldehyde-3-phosphate dehydrogenase |
| GPDA | Glycerol-3-phosphate dehydrogenase [NAD(+)], cytoplasmic |
| GUAD | Guanine deaminase |
| H15 | Histone H1.5 |
| H2AJ | Histone H2A.J |
| HB2B | Rano class II histocompatibility antigen, B-1 beta chain |
| HBA | Hemoglobin subunit alpha-1/2 |
| HBB1 | Hemoglobin subunit beta-1 |
| HCD2 | 3-hydroxyacyl-CoA dehydrogenase type-2 |
| HEMO | Hemopexin |
| HMGA1 | High mobility group protein HMG-1 / HMG |
| HNRH2 | Heterogeneous nuclear ribonucleoprotein 2 |
| HNRPM | Heterogeneous nuclear ribonucleoprotein M |
| HSPB1 | Heat shock protein beta-1 |
| IF2A | Eukaryotic translation initiation factor 2 subunit 1 |
| IF4H | Eukaryotic translation initiation factor 4H |
| IF6 | Eukaryotic translation initiation factor 6 |
| IFM3 | Interferon-induced transmembrane protein 3 |
| K2C6A | Keratin, type II cytoskeletal 6A |
| KPYM | Pyruvate kinase |
| LDHA | L-lactate dehydrogenase A chain |
| LEG3 | Galectin-3 |
| LMNA | Prelamin-A/C |
| MDHM | Malate dehydrogenase, mitochondrial |
| METK2 | S-adenosylmethionine synthase isoform type-2 |
| ML12B | Myosin regulatory light chain 12B |
| MOES | Moesin |
| MYH9 | Myosin 9 |
| MYH10 | Myosin-10 |
| MYH11 | Myosin-11 |
| MYL6 | Myosin light polypeptide 6 |
| MYO1C | Unconventional myosin 1C |
| NCAM1 | Neural cell adhesion molecule 1 |
| NDRG1 | Protein NDRG1 |
| NEDD4 | E3 ubiquitin-protein ligase NEDD4 |
| NHRF1 | Na(+)/H(+) exchange regulatory cofactor NHE-RF1 |
| NUP53 | Nucleoporin |
| OAT | Ornithine aminotransferase, mitochondrial |
| P4HA1 | Prolyl 4-hydroxylase subunit alpha-1 |
| PA1B2 | Platelet-activating factor acetylhydrolase IB subunit β |
| PDIA4 | Protein disulfide-isomerase A4 |
| PGK1 | Phosphoglycerate kinase 1 |
| PHB | Prohibitin |
| PHB2 | Prohibitin-2 |
| PLEC | Pleckstrin homology-like domain family B member 1 (Fragment) |
| PLP2 | Proteolipid protein 2 |
| PP1G | Serine/threonine-protein phosphatase PP1-gamma catalytic subunit |
| PRDX2 | Peroxiredoxin-2 |
| PRDX6 | Peroxiredoxin-6 |
| PROF1 | Profilin-1 |
| PRS7 | 26S protease regulatory subunit 7 |
| PSB10 | Proteasome subunit beta type-10 |
| PTGIS | Prostacyclin synthase |
| PTRF | Polymerase I and transcript release factor |
| QKI | Protein quaking |
| RAB2A | Ras-related protein Rab-2A |
| RACK1 | Receptor of activated protein C kinase 1 |
| RB11B | Ras-related protein Rab-11B |
| RL34 | 60S ribosomal protein L34 |
| RMD3 | Regulator of microtubule dynamics protein 3 |
| RRAS | Ras-related protein R-Ras |
| RS14 | 40S ribosomal protein S14 |
| RS17 | 40S ribosomal protein S17 |
| RS18 | 40S ribosomal protein S18 |
| RS20 | 40S ribosomal protein S20 |
| 02-Sept | Septin-2 |
| SAHH | Adenosylhomocysteinase |
| SBP1 | Single-stranded DNA-binding protein, mitochondrial |
| SCPDL | Saccharopine dehydrogenase-like oxidoreductase |
| SPTN1 | Spectrin alpha chain, non-erythrocytic 1 |
| S10A4 | Protein S100-A4 |
| S10A6 | Protein S100-A6 |
| S10AA | Protein S100-A10 |
| SYUG | Gamma-synuclein |
| TAGL | Transgelin |
| TAGL2 | Transgelin-2 |
| TAP2 | Antigen peptide transporter |
| TBB2A | Tubulin beta-2A chain |
| TBCA | Tubulin-specific chaperone A |
| TPM3 | Tropomyosin alpha-3 chain |
| UB2V2 | Ubiquitin-conjugating enzyme E2 variant 2 |
| UBA1 | Ubiquitin-like modifier-activating enzyme 1 |
| UGGG1 | UDP-glucose : glycoprotein glucosyltransferase 1 |
| VAMP8 | Vesicle-associated membrane protein 8 |
| VAT1 | Synaptic vesicle membrane protein VAT-1 |
| VTDB | Vitamin D-binding protein |
| WDR81 | WD repeat-containing protein 81 |
